# Advancing prevention science for maltreatment exposed children: predicting mental health symptoms with a combined neurocognitive vulnerability index

**DOI:** 10.1007/s00787-025-02838-y

**Published:** 2025-10-22

**Authors:** Mattia I. Gerin, Essi Viding, Diana J. N. Armbruster‑Genç, Jonathan P. Roiser, Eamon J. McCrory

**Affiliations:** 1 Escuela de Psicología, Pontificia Universidad Católica de Chile, Santiago, Chile; 2 Division of Psychology and Language Sciences, University College London, 26 Bedford Way, London, WC1H 0AP UK; 3 Anna Freud, London, UK; 4 Department of Psychology, RPTU Kaiserslautern-Landau, Landau, Germany; 5 Institute of Cognitive Neuroscience, University College London, London, UK

## Abstract

Childhood maltreatment is an established risk factor for poor mental health, yet not all maltreatment-exposed children develop psychiatric symptoms. This highlights the need for individualised metrics to detect those at heightened risk. Previous efforts to develop such metrics have largely focused on maltreatment severity and aggregate demographic risk. Although neurocognitive alterations in domains like social cognition and reward processing have been linked to maltreatment, most research has examined cross-sectional group-level differences in isolated domains. This proof-of-concept study examines whether indexing combined neurocognitive vulnerability can help predict which maltreatment-exposed children are most at risk of future symptom increases. A sample (mean age = 12.8) of maltreatment-exposed children (MT = 85) and propensity-score matched (PSM) non-maltreated peers (NMT = 90) were assessed at baseline using five neurocognitive tasks spanning various domains of social cognition and reward processing. A combined neurocognitive vulnerability metric was created, and symptom changes were measured 1.5 years later for a subset of individuals (MT = 57, NMT = 47). Combined neurocognitive vulnerability predicted future mental health symptom increases in the maltreatment-exposed group, even after controlling for baseline symptoms and clinical status. Maltreatment-exposed children in the high neurocognitive vulnerability category had a 92.1% adjusted predicted probability of experiencing worsening symptoms over time. Furthermore, we found that children in the maltreatment-exposed group were more likely to fall into medium and high neurocognitive vulnerability categories than non-maltreated peers. These preliminary findings underscore the potential of using combined neurocognitive vulnerability metrics to identify maltreatment-exposed children at the increased risk for future mental health problems, supporting its role in prevention strategies.

**Supplementary Information:**

The online version contains supplementary material available at 10.1007/s00787-025-02838-y.

## Introduction

Childhood maltreatment – including physical, sexual, and emotional abuse, exposure to domestic violence, and neglect – has long been recognised as a significant predictor of poor mental health across the lifespan [1, 2]. Recent quasi-experimental [3] and genetically informed [4] studies further suggest a causal link. Early adversity is also recognised as a critical environmental factor contributing to a range of neurocognitive alterations thought to underlie latent psychiatric vulnerability [5]. However, the relationship between maltreatment and psychopathology is not deterministic; many individuals demonstrate resilient outcomes, and when mental health problems do emerge, they often do so gradually rather than immediately after early trauma [6]. Thus, it is crucial to seize this window of opportunity – before psychopathology emerges, becomes entrenched, and harder to treat – by delivering preventative interventions that can offset risk trajectories [7].

The prevention and promotion of mental health is increasingly recognised not only as an ethical imperative but also as an economic one [8]. A key requirement for developing cost-effective preventative approaches is the timely identification of children and young people at heightened latent psychiatric vulnerability. However, we currently lack reliable prognostic tools for this purpose. Recent progress has come from studies demonstrating the feasibility of computing an individualised vulnerability metric for those exposed to early adversity [9, 10]. This metric was shown to predict future mental health and functional outcomes by combining demographic, intellectual ability, and psychosocial factors. To build upon these findings, it is crucial to investigate additional predictors that could help identify latent psychiatric vulnerability on an individual basis.

A promising direction for development is the examination of the prognostic value of neurocognitive indices. A growing body of empirical research indicates that childhood maltreatment is associated with system-level neurocognitive recalibration across various domains, including social cognition (e.g., threat and affect detection) and reward processing (e.g., contingency-based learning), even before overt clinical symptoms emerge [5, 11]. While most existing studies have utilised cross-sectional designs, preliminary longitudinal data suggest that these neurocognitive alterations can predict future mental health outcomes [12, 13]. However, much of the research has focused on isolated domains that can detect variability at a group level. An individualised vulnerability metric based on a range of neurocognitive domains, rather than single metrics, has the potential to improve the identification of those at greater latent psychiatric vulnerability and reveal potential risk mechanisms to be targeted through preventative intervention.

To our knowledge, current efforts to develop individualised psychiatric vulnerability screening tools have primarily focused on quantifying the cumulative number of early adversities as a proxy for trauma severity [14, 15], or, more recently, on indexing demographic, psychosocial, and intellectual functioning risk factors [9]. Among children and young people not specifically selected for maltreatment exposure, additive vulnerability metrics have been explored by aggregating measures of maladaptive cognitive styles (e.g., perceived self-efficacy, rigid and perfectionistic beliefs, perceived locus of control, and rumination). These aggregated measures have been shown to predict the onset of depressive symptoms over time [16–18]. However, in both the early adversity and general developmental psychopathology literature, a combined vulnerability metric based on alterations in neurocognitive functioning across several domains has yet to be examined as a potential index of latent psychiatric risk.

Given these gaps in the literature, the primary aim of this proof-of-concept study is to examine whether a combined metric of neurocognitive functioning can identify maltreatment-exposed children and young people at greater psychiatric vulnerability. Specifically, we seek to determine if co-occurring neurocognitive alterations, in domains of social cognition and reward processing, can predict symptom increases over time (1.5 years after baseline assessment). To this end, we recruited a baseline sample of 175 children and adolescents (mean age = 12.8), including a group with substantiated maltreatment experiences (*n* = 85) and a propensity-score matched group of non-maltreated peers (*n* = 90). A subsample (*n* = 98) had longitudinal mental health data available and formed the primary sample for predictive analyses. At baseline, participants completed computerised neurocognitive tasks assessing various domains of social cognition and reward processing, including trust attributions to unfamiliar faces, emotional intensity perception, attentional biases for threat-related social cues, effort-based reward sensitivity, and contingency-based learning in volatile contexts. Performance on each task was classified as either normative or atypical to compute an aggregate neurocognitive vulnerability metric (i.e., low, medium, or high). Consistent with longitudinal studies that have examined neurocognitive domains in isolation [12, 13, 19] our primary prediction was that an atypical neurocognitive performance across multiple domains would be predictive of future increases in symptoms. We also expected a higher prevalence of atypical neurocognitive profiles in the maltreatment-exposed group, in line with prior work on maltreatment-related neurocognitive recalibrations [5, 20].

## Methods

### Participants

At baseline, 175 children and adolescents (mean age = 12.8) participated in this study (Table 1). Participants who had experienced maltreatment requiring statutory intervention comprised the maltreatment exposed group (MT = 85) and were recruited via London Social Services departments. The parent, legal guardian and/or social work professional were asked to assess current safety and stability of placement before providing consent for any child or young person to participate. Peers with no prior Social Services contact, comprising the non-maltreatment exposed group (NMT = 90), were recruited through schools in similar areas to match the MT group on demographic variables, including socioeconomic status, ethnicity, age, pubertal status, sex, and IQ. Exclusion criteria included a pervasive developmental disorder, neurological abnormalities, and an IQ below 70. Table 1 presents an overview of the group’s baseline demographics, cognitive abilities, psychological functioning. At follow-up, 98 participants (MT = 57, NMT = 47) had available symptoms data (see Table S2).

**Table 1:** Demographic variables, cognitive ability, and psychiatric symptoms in maltreatment-exposed (MT; *n* = 85) and non-maltreated exposed participants (NMT; *n* = 90)

Measures		MT	NMT
		Percentage	Percentage
Gender (Female)		48.2%	52.2%
Ethnicity (Caucasian)		38.8%	44.4%
		Mean (SD)	Mean (SD)
Age ^a^		12.8 (2.4)	12.9 (2.1)
Pubertal status ^b^		2.4 (0.8)	2.4 (0.8)
WASI-IQ ^c^*		98.5 (11.4)	104.4 (11.0)
Socio-economic status ^d^*		3.3 (1.0)	2.9 (1.0)
SDQ total score ^e^*		12.4 (6.4)	7.3 (5.6)

* *p* <.05

^a^Age range: 8–16 years. ^b^ Pubertal status was measured with the Puberty Development Scale (PDS) [21]; when available a composite score of both parent/carer and child reports was used (MT = 65, NMT = 68); 19 MT and 23 NMT participants had either the parent/carer or child report available; 2 NMT and 2 MT participants’ scores were imputed. ^c^ WASI-IQ: two IQ-subscales derived from the Wechsler Abbreviated Scales of Intelligence [22]. ^d^ Socio-economic status was measured using the highest parental/carer’s level of education rated on a 6-point scale from 0 (no formal qualifications) to 5 (postgraduate qualifications); 2 NMT and 4 MT participant’s scores were imputed. ^e^ SDQ: Strength and Difficulties Questionnaire; MT 83 and NMT 74 had available baseline SDQ data [23]

### Measures

#### Combined neurocognitive vulnerability 

A combined neurocognitive vulnerability metric was developed based on participants’ baseline performance in five behavioural experimental paradigms that assess neurocognitive functioning across domains of social cognition and reward processing that have previously been associated with maltreatment exposure [6, 24, 25]. The paradigms included: (i) a ‘Dot-Probe Task’ for the assessment of attention bias towards threat-related facial expressions; (ii) a trustworthiness face-judgement task to assess trust attributions towards unfamiliar faces; (iii) an emotion recognition task to assess the perceived emotional intensity of dynamic facial expressions; (iv) the ‘Environmental Reward Learning Task’ to measure the ability to adjust contingency-based learning rates in stable and volatile environments; and (v) the ‘Apple Gathering Task’ to assess effort-based reward sensitivity. Detailed descriptions of these five paradigms, and the metric extracted from each task, are provided in the supplementary material and in published research [12, 19, 26, 27].

Participants were included if they had available data on at least two of the five tasks (see Supplementary Table S5). Each participant’s performance on a task was categorized as normative (within 1 SD from the sample’s overall mean – i.e. MT and NMT groups combined) or atypical (see supplementary materials subsection ‘Cumulative Neurocognitive Vulnerability Metric’ for task-specific rationale and classification directionality, including cases where deviations in both directions were considered atypical). Subsequently, a trinary combined neurocognitive vulnerability metric was computed. As can been seen in Table 2, participants with a normative score on all available tasks were classified as low-vulnerability (0); those with an atypical score on only one task were classified as medium- vulnerability (1); and those with atypical scores on two or more tasks were classified as high-vulnerability (2). The supplementary materials document provides a detailed description of how the neurocognitive vulnerability metric was computed.

**Table 2:** Statistically significant distribution of cumulative neurocognitive vulnerability levels (low, medium, and high) by maltreatment exposed (MT) and non-maltreatment exposed (NMT) groups

	Low vulnerability	Medium vulnerability	High vulnerability
NMT group	52 (57.8%)	29 (32.2%)	9 (10.0%)
MT group	34 (40.0%)	36 (42.4%)	15 (17.6%)

Percentage values are expressed as a proportion of the row totals. Of the 24 participants in the High-risk group, 18 had atypical score on two tasks and 6 had atypical scores on three tasks. The association between group and cumulative neurocognitive vulnerability level was statistically significant (χ² 5.9, bias 1.8, SE 5.1, *p* <.05 (95% CI 0.3,16.6), bootstrapped *R* 5000)

#### Symptom change

Overall symptoms were assessed at baseline and at follow-up (approximately 1.5 years later later) using the caregiver’s reports on the Strengths and Difficulties Questionnaires (SDQ). This is a well-established and widely used measure with sound psychometric properties, including high internal consistency (mean Cronbach a = 0.73) and good test-retest reliability (mean correlation = 0.62) [28]. To index early shifts in emotional and behavioural functioning, we created a binary outcome variable indicating whether SDQ total scores increased from baseline to follow-up (1 = increase; 0 = no increase or stable/decreased). This approach was selected to align with the study’s central aim of detecting early signs of symptom escalation that may indicate increased risk — prior to the emergence of clinically significant psychopathology. Baseline SDQ scores were included as a covariate in all regression models to statistically adjust for initial symptom severity, thereby isolating predictors of longitudinal change. This strategy supports the broader goal of contributing to early risk detection and prevention science by identifying children who may be on worsening mental health trajectories.

### Procedures

The data used in this study was collected as part of a larger ESRC/NSPCC funded study (539220). The child’s legal guardian provided written consent. All children provided written and verbal assent. The UCL Research Ethics Committee approved the procedures implemented in this study (11767/001). During an initial Home visit, both the participant and a parent or carer completed the demographic information, symptoms questionnaire, and psychometric testing. The experimental tasks were completed during a second session. The symptoms follow-up assessment took place, on average, 19 months after the baseline assessment (mean_days_ = 580.0, SD_days_ = 48.6, approximately 1.5 months).

### Data analysis

#### Combined neurocognitive vulnerability and longitudinal symptoms change

We performed a bootstrapped logistic regression using participants with available longitudinal symptoms data (MT = 57, NMT = 41; see supplementary Table S2 for the longitudinal subsample characteristics) to examine if combined neurocognitive vulnerability was predictive of future symptoms change (increase vs. decrease/stable). Baseline symptom levels (SDQ total score) were included as a covariate in all regression models to statistically adjust for initial symptom severity.

To complement the logistic regression analyses, we calculated sensitivity, specificity, and positive predictive value (PPV) for the high neurocognitive vulnerability group within the MT sample. Sensitivity was defined as the proportion of participants with symptom increase who were classified as high-vulnerability; specificity was the proportion of those with stable/decreasing symptoms who were classified as low or medium vulnerability; and PPV was the proportion of high-vulnerability individuals who experienced symptom increase. These metrics were calculated using raw counts presented in Supplementary Table S7.

#### Childhood maltreatment and combined neurocognitive vulnerability

A Pearson’s Chi-squared test with bootstrapping was conducted to examine the relationship between maltreatment group status (MT and NMT) and combined neurocognitive vulnerability levels. The p-value was estimated using 5000 simulations due to the presence of low frequencies in some cells. This approach ensures a more accurate p-value estimate compared to the asymptotic Chi-squared test.

#### Propensity score matching (PSM)

Due to the statistically significant group difference on demographic variables, such as IQ and SES (Table 1), PSM was performed to mitigate the influence of potential confounding variables on group differences [29–31]. PSM can manage numerous control variables effectively, avoiding issues of model over-fitting or multicollinearity that often complicate the evaluation of predictor variables’ effects on outcome variables in conventional inferential statistical models [29, 32, 33]. Importantly, PSM is robust even for variables with significant distribution differences across groups, unlike traditional covariate adjustment methods. The MT group was propensity-score matched to the NMT group for the following variables: age, pubertal status, gender, socio-economic status, IQ, ethnicity (See Table S1 in the supplementary document). ‘Full matching’ achieved satisfactory results (see Table S1 and the supplementary document for a detailed description of the PSM procedure).

## Results

### Combined neurocognitive vulnerability and longitudinal symptoms change

**Fig. 1:**
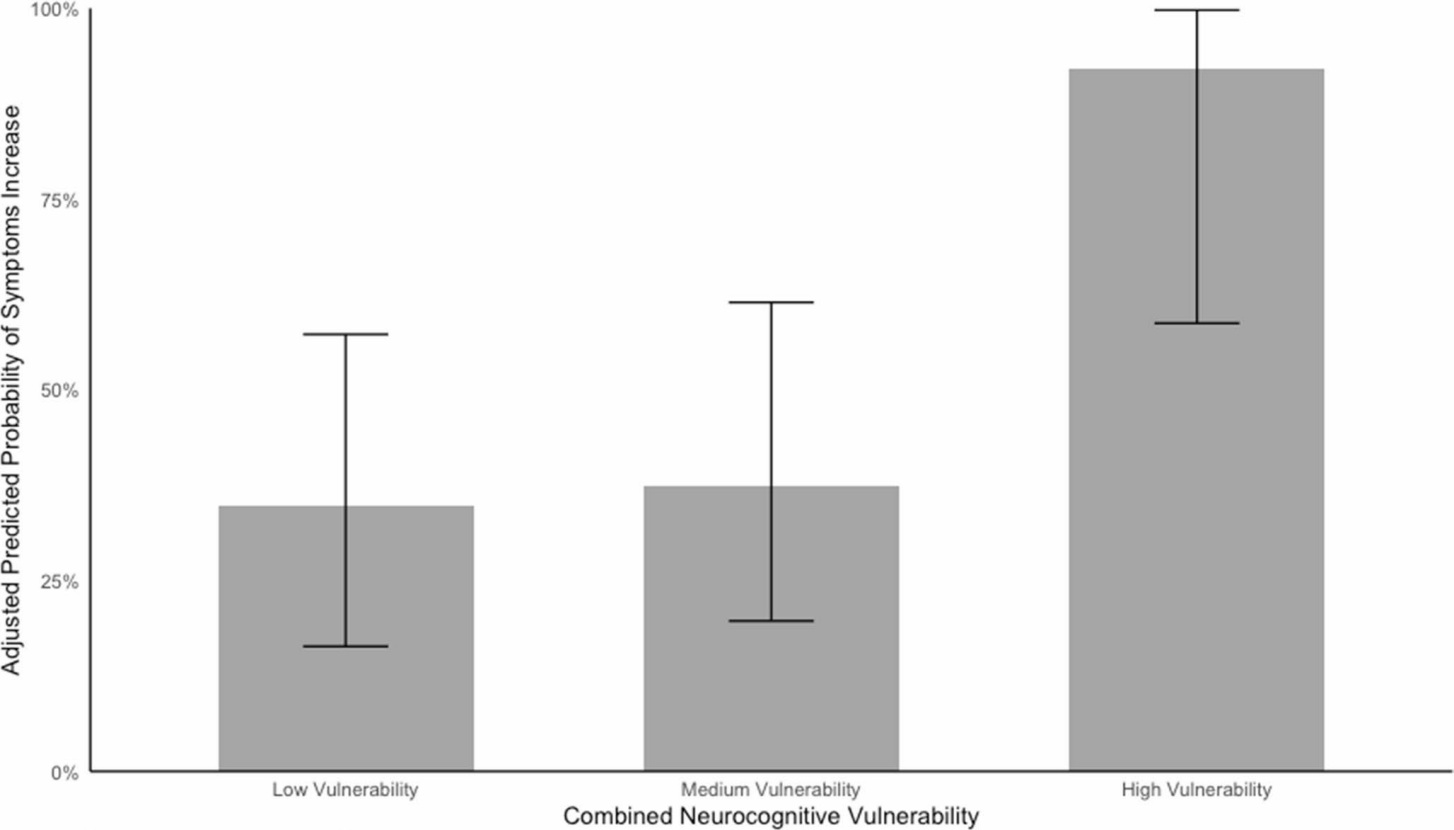
Predicted probability of longitudinal symptoms increase by neurocognitive vulnerability categories (low, medium and high) in maltreatment-exposed participants (MT group). Note: Error bars represent 95% Clopper-Pearson confidence intervals within each vulnerability category. The Predicted Probability values are adjusted for baseline symptom levels. Low Vulnerability (*n* = 23), Medium Vulnerability (*n* = 23), High Vulnerability (*n* = 11)

Within the MT group with available longitudinal data (MT = 57), the logistic regression bootstrapped results (*R* = 5000) indicated that being in the neurocognitive high-vulnerability category, compared to those with low-vulnerability (the reference category), was associated with a large and significant greater likelihood of experiencing an increase in symptoms at follow-up, B = 3.1; OR = 21.8, *p* <.05 (95% CI = 1.3, 20.7). The baseline symptoms covariate, B = −0.1; OR = 0.9, *p* >.05 (95% CI = −0.2, 0.1), was not predictive of longitudinal symptom change (see the full regression model in Table 3). Furthermore, sensitivity analyses (reported in the supplementary materials, Table S8) showed that the combined neurocognitive high-vulnerability metric remained a significant predictor of future symptoms increase, even after excluding eight participants who presented with unusually high symptoms at baseline (SDQ total scores ≥ 20). In summary, the data suggest that being in the neurocognitive high-vulnerability category following maltreatment exposure is predictive of future increase in symptoms, even after controlling for baseline symptoms levels or clinical status. On the other hand, in the NMT group (Fig. 1S), combined neurocognitive vulnerability scores were not significantly associated with symptom change over time (for the contingency table and full regression model see, respectively, supplementary Tables S9 and Table S10).

**Table 3:** Logistic regression results predicting symptoms change (decrease/stable vs. increase) from cumulative neurocognitive vulnerability levels (low, medium and high) and baseline symptom levels (SDQ baseline total score) in the MT group

	B	Bias	SE	95% CI	Odds Ratio (OR)
Intercept	0.0	0.1	−0.8	[−1.6, 1.8]	1.0
Medium vulnerability (ref: low vulnerability)	0.1	−0.0	0.8	[−1.3, 1.6]	1.1
High vulnerability (ref: low vulnerability) *	3.1	5.9	8.0	[1.3, 20.7]	21.8
Baseline Symptoms	−0.1	−0.0	−0.1	[−0.2, 0.1]	0.9

*sig. bootstrapped predictors

Cumulative neurocognitive vulnerability is an ordinal variable with three levels: low, medium and high. The low-vulnerability category serves as the reference group. Results are obtained using bootstrapping with 5000 repetitions. The coefficients (B) represent the log odds. Bias is the difference between the bootstrapped estimate and the original coefficient

#### Sensitivity, Specificity, and Predictive Value

Among maltreatment-exposed participants (Fig. 1; see Supplementary Table S7), we evaluated the utility of the high neurocognitive vulnerability classification in predicting longitudinal symptom increase. Sensitivity was defined as the proportion of individuals who experienced symptom increase and were correctly identified as high-vulnerability (10/27; 37.0%). Specificity was defined as the proportion of individuals with stable or decreasing symptoms who were correctly classified as low or medium vulnerability (29/30; 96.7%). Finally, the positive predictive value (PPV) of the high-vulnerability category was 90.9% (10/11), indicating that nearly all individuals categorised as high-vulnerability did in fact experience worsening symptoms over time. This suggests that while the classification is highly specific and has high PPV, sensitivity is lower—highlighting that high neurocognitive vulnerability may signal a particularly high-risk subgroup, but not all individuals who deteriorate are captured by this profile.

#### Childhood maltreatment and combined neurocognitive vulnerability

The chi-square test bootstrap results (*R* = 5000) indicated a statistically significant difference in the distribution of combined neurocognitive vulnerability scores (low-, medium-, and high-vulnerability) between children with (MT group) and those without (NMT group) a history of maltreatment, χ² = 5.9, bias = 1.8, SE = 5.1, *p* <.05 (95% CI = 0.3,16.6). Specifically, as expected, children with maltreatment exposure, compared to their non-maltreated peers, had a higher proportion of participants in the medium- (OR = 1.24) and high-vulnerability categories (OR = 1.67), and a lower proportion in the low- vulnerability category (OR = 0.65) (see Table 2). After a stringent correction for IQ and demographic variables using PSM (see details in the supplementary Table S1), statistically significant group differences in the distribution of combined neurocognitive vulnerability scores remained between children with and without a history of maltreatment, χ² = 3.7, bias = 1.1, SE = 3.0 *p* <.05 (95% CI = 0.3, 9.8) (see contingency Table S4). In other words, independently of demographic and cognitive variables, individuals in the maltreatment exposed group, compared to non-exposed peers, had a higher probability of being in the medium or high combined neurocognitive vulnerability categories.

## Discussion

In the context of increased emphasis on mental health prevention, the aim of the current longitudinal study was to examine whether a range of neurocognitive probes could be used to detect psychiatric vulnerability among maltreatment-exposed children. There were two main results. First, we found that a combined neurocognitive vulnerability metric, spanning various domains of social cognition and reward processing, predicted future symptom increases in maltreatment-exposed children. Even after controlling for baseline symptoms and clinical status, children classified as high-vulnerability (i.e. with atypical functioning in two or three neurocognitive domains) were substantially more likely to present with worsening mental health symptoms at follow-up 1.5 years later. Second, children with maltreatment exposure, compared to propensity-score matched non-maltreated peers, had higher combined neurocognitive vulnerability scores. That is, they were more likely to exhibit atypical neurocognitive functioning across multiple domains.

Building upon prior efforts, this study introduces a fresh approach for assessing latent psychiatric risk in maltreatment-exposed children, before frank psychopathology has emerged. Prior research that has sought to identify those who may be most vulnerable predominantly focused on the severity of maltreatment, such as the number of adverse childhood experiences [14, 15], isolated neurocognitive domains [34–36], or aggregate indices of demographic, intellectual functioning, and psychosocial risk [9]. As we move more towards a model of secondary prevention the timely identification of children at increased psychiatric vulnerability is essential to ensure support and intervention during this critical period – before symptoms emerge, become ingrained, and are thus harder to treat. We know that maltreatment-exposed individuals who develop mental health problems often have a poorer prognosis and can respond less favourably to treatments [37–39]. Therefore, accurate early identification is both an ethical and economic necessity [8]. Our preliminary findings suggest that integrating neurocognitive probes across multiple domains offers a promising avenue for adding clinically meaningful information to *individualised* risk assessments. This approach may also help identify possible aetiological mechanisms that could be prioritised in any preventative intervention aimed at fostering resilient outcomes.

Another key novel contribution of this study is that a cumulative measure, encompassing multiple domains of neurocognitive functioning, may be feasible and effective for detecting maltreatment-related recalibrations. This is consistent with the view that maltreatment experiences result in system-level neurocognitive alterations across multiple domains [5, 20]. Crucially, the group difference in the neurocognitive vulnerability metric was independent of potentially confounding demographic or cognitive factors that were stringently controlled for using PSM. This finding, while requiring replication, highlights the potential for three important lines of future enquiry. First, indexing a constellation of concurrent domains in future research may help identify which neurocognitive subdomains are more strongly associated with specific outcomes (e.g., internalising vs. externalising symptom clusters, difficulties in educational settings, poor interpersonal functioning). Second, this multiple-domain approach may also motivate new research that can contribute to current debates about how different dimensions of childhood maltreatment – such as the developmental timing of exposure [40, 41], subjective appraisal of early trauma [42], and types or number of early adverse events [43, 44] – may contribute to specific neurobiological and psychological outcomes. Third, and perhaps most tentatively, our findings raise the possibility that it may not be the degree of atypicality within any single neurocognitive domain that best signals psychiatric vulnerability, but rather the accumulation of alterations across multiple domains. In this sample, children exhibiting atypical performance in more than two domains were at greatest risk of symptom increases over time. While replication is needed, this cumulative pattern may reflect system-level disruptions with functional consequences that are not easily captured when focusing on isolated constructs. Future work should therefore examine whether multi-domain profiles outperform dimensional scores from any one task, and explore the potential existence of a latent neurocognitive risk factor using larger samples and multivariate analytic approaches. This should include comparisons with more conventional aggregation methods, such as z-score-based composites or weighted continuous scores.

Given the preliminary nature of this study, several important limitations must be acknowledged when interpreting the results and planning future research. First, we examined a limited range of neurocognitive domains. This study should be expanded to include additional domains associated with maltreatment exposure and known to be potential mechanisms of latent vulnerability [5, 24, 45]. We would suggest that this should encompass, but not be limited to: executive functions critical for effective interpersonal interactions, such as emotional regulation; interpersonal problem-solving delay discounting; and declarative memory, including autobiographical memory. Second, the current sample size limited our ability to employ more complex multivariate analytic approaches, such as confirmatory factor analysis and machine learning techniques. This limitation is particularly relevant for the high-vulnerability subgroup, which included a relatively small number of participants, and therefore findings relating to this category should be interpreted with caution. These methods, facilitated by a large sample, could provide more fine-grained insights into the existence of the proposed ‘latent’ theoretical construct (i.e., maltreatment-related cumulative neurocognitive vulnerability) and help identify the most relevant and potent predictors of psychiatric vulnerability. 

Third, while our use of a binary outcome (symptom increase vs. stable/decrease) aligns with the study’s preventative aims, we acknowledge that this approach does not capture the magnitude or clinical meaningfulness of symptom change. Our primary goal was to identify children who may be on a worsening trajectory — before symptom escalation reaches diagnostic thresholds. However, this method has limitations. Symptom change was operationalised using caregiver-reported SDQ scores, which may not fully capture difficulties across all domains or do so equally across groups, particularly when caregivers differ in their relationship to the child (e.g., parent vs. professional carer). Future studies should therefore incorporate multiple-informant assessment approaches to improve the robustness and comparability of outcome measurement. Additionally, longer follow-up intervals and more frequent symptom assessments would enable testing whether cumulative neurocognitive vulnerability also predicts the onset of diagnosable psychiatric disorders or more substantial clinical deterioration. Such extensions would help clarify the prognostic value of this metric across different stages of vulnerability and disorder emergence. We also recognise that future studies should formally compare modelling strategies — such as treating neurocognitive predictors and symptom change as continuous versus categorical — to determine which approach most effectively captures cumulative neurocognitive vulnerability and its clinical relevance. Finally, there is growing recognition that the impact of maltreatment-related neurocognitive recalibrations in shaping future outcomes is modulated by domains of interpersonal functioning, such as the presence of adaptive or maladaptive support networks, social competence and confidence [6]. Identifying such social factors in future research could be leveraged to increase the translational relevance of these findings, directly informing the development of evidence-based preventative interventions that could foster adaptive interpersonal functioning. Fourth, follow-up data were available for only a subset of participants, due to study design constraints. Nonetheless, comparisons across baseline characteristics revealed no strong or systematic differences between participants with and without follow-up, suggesting that attrition is unlikely to have introduced substantial bias (see Supplementary Table S2).

Despite these limitations, this study has several notable strengths. First, it is the first to apply a combined neurocognitive vulnerability metric in a maltreatment-exposed sample, offering a novel experimental approach to predicting psychiatric vulnerability in this high-risk population. By focusing on prediction, the study provides a valuable tool for identifying children most at risk for future mental health problems. Second, the recruitment of a sample with substantiated maltreatment exposure adds robustness to the findings, ensuring that the observed relationships are grounded in well-documented cases of adversity. Finally, the longitudinal design enables the tracking of changes in symptoms over time, providing a more comprehensive and nuanced view of risk trajectories compared to most neurocognitive studies of childhood maltreatment, which typically employ a cross-sectional design.

In conclusion, this study introduces a novel cumulative neurocognitive vulnerability index that simultaneously captures alterations across multiple domains of neurocognitive functioning. The results suggest that this assessment method may hold significant implications for prevention science, particularly in identifying children at heightened psychiatric risk following maltreatment exposure. Developing cost-effective screening tools is a crucial stepping stone, as these tools are necessary for determining who should be prioritised when deploying indicated preventative approaches. Only by accurately identifying those at greatest vulnerability can we design affordable, scalable approaches that effectively offset risk trajectories and reduce the mental health impact of childhood maltreatment.

## Supplementary Information

Below is the link to the electronic supplementary material.

Supplementary file1 (DOCX 96.4 KB)

## Data Availability

No datasets were generated or analysed during the current study.
