# Supplementary material for: Advancing prevention science for maltreatment exposed children: predicting mental health symptoms with a combined neurocognitive vulnerability index
Source: Eur Child Adolesc Psychiatry. 2025 Oct 22;35(3):835–43. doi: 10.1007/s00787-025-02838-y (PMC13212408; doi:10.1007/s00787-025-02838-y)
Supplement: Supplementary file 1 — Supplementary file1 (DOCX 96.4 KB) [file 787_2025_2838_MOESM1_ESM.docx]

**Supplementary Document**

**Advancing prevention science for maltreatment exposed children: Predicting mental health symptoms with a combined neurocognitive vulnerability index**

Mattia I. Gerin^1,2,3^; Essi Viding^2^; Diana J. N. Armbruster‑Genç^2,4^; Jonathan P. Roiser^5^ & Eamon J. McCrory^2,3,*^

*^1^ Escuela de Psicología, Pontificia Universidad Católica de Chile, Santiago, Chile; ^2^ Division of Psychology and Language Sciences, University College London, London, UK; ^3^ Anna Freud Centre, London, UK; ^4^ Department of Psychology, RPTU Kaiserslautern-Landau, Landau, Germany; ^5^ Institute of Cognitive Neuroscience, University College London, London, United Kingdom*

*^*^Address Correspondence to this author at Division of Psychology and Language Sciences, University College London, 26 Bedford Way, London WC1H 0AP, UK; Email:* [e.mccrory@ucl.ac.uk](mailto:e.mccrory@ucl.ac.uk).

**Supplementary Methods**

**Propensity Score Matching (PSM)**

The influence of potential confounding variables can be mitigated through Propensity Score Matching (PSM). This encompasses a variety of statistical methods that are utilised prior to any inferential statistical analysis to balance the distribution of covariates between treatment and control groups [1]. The most used matching procedures include exact pairing, weighting, or sub-classification, or a combination of these approaches. PSM can handle many control variables without leading to model over-fitting or multicollinearity issues, which can hinder the assessment of the impact of predictor variables on dependent variables in traditional regression modelling. Notably, unlike traditional covariate adjustment methods, PSM is also reliable for variables with substantial distribution differences across groups [2–5].

When using PSM, it is important to examine various matching methodologies to determine which one achieves the greatest reduction in distance (i.e. distribution of covariates) between the control and treatment groups [2–4] The effectiveness of the matching process (i.e. the balance between the two groups after matching) is often evaluated using a standardised mean difference (i.e. effect size) of the propensity score [1, 2]. Although there is no consensus on a specific cut-off, it has been suggested that values between 0.1 and 0.25 are acceptable thresholds for standardised mean differences post-matching [6]. Additionally, standardised mean differences for each variable before and after matching can be interpreted as effect sizes, with values below 0.2 indicating a small difference, around 0.5 a medium difference, and 0.8 a large difference [1, 5].

In this study, the R software package MatchIT [2] was used to implement four PSM methods utilizing different algorithms to match participants. Nearest Matching and Optimal Matching are similar in that they use a 1:1 matching approach. In Nearest Matching, for each participant in the treatment group, one or more participants with the closest propensity score (i.e. smallest distance) are selected from the control group. Optimal Matching performs 1:1 matching to minimize the overall distance between the two groups. Additionally, Genetic Matching and Full Matching, which allow for flexible matching within subclasses through weighting, were explored. In this study, the following potential confounding variables were selected for PSM between the treatment and control groups: age, gender, IQ, ethnicity, and socio-economic status (measured by parental level of education).

***PSM Outcome*.** Full Matching allowed for the best reduction in the overall standardised mean difference across all covariates (from 0.19 to 0.09 post-matching - Table S1). For example, the standardized mean difference for IQ and socio-economic status (i.e. the two variables with the largest absolute standardised mean difference before matching) decreased from, respectively, -0.52 to -0.07 and from 0.38 to 0.05 (Table S1). Furthermore, post-matching the standardised mean difference for each variable was below the small effect size threshold (i.e. < 0.2), with absolute values ranging between 0.05 and 0.16.

| **Table S1**  *Balance in covariates before and after propensity score matching (PSM) between the maltreatment-exposed (MT; n=85) and non-maltreated exposed participants (NMT; n=95)* | | | | | | |
| --- | --- | --- | --- | --- | --- | --- |
|  | Means / Percentages | | | | Standardized Mean Difference^1^ | |
|  | MT | NMT | NMT *Post* Matching | *Before* Matching | | *Post* Matching |
| Distance | 0.5 | 0.4 | 0.5 | 0.68 | | 0.03 |
| Age | 12.8 | 12.9 | 12.6 | -0.03 | | 0.07 |
| Pubertal status | 2.4 | 2.4 | 2.4 | 0.00 | | -0.05 |
| WASI-IQ | 98.5 | 104.4 | 99.2 | -0.52 | | -0.07 |
| Socio-economic status | 3.3 | 2.9 | 3.3 | 0.38 | | 0.05 |
| Gender (Female) | 48.2% | 52.2% | 54.5% | -0.08 | | -0.12 |
| Ethnicity (Caucasian) | 38.8% | 44.4% | 31.0% | -0.12 | | 0.16 |
| Average Absolute Standardized  Mean Difference^2^ | - | - | - | 0.19 | | 0.09 |
| MT = Maltreated group (n =85), Non-MT = Non-Maltreated group (n=90), CT = Control group.  ^1^ Each standardized mean difference is obtained by subtracting the mean in the MT group minus mean in the CT group, divided by the standard deviation of the MT group. ^2^ The average absolute standardized mean difference is the average of the absolute values of standardized mean differences for all covariates. | | | | | | |

**Longitudinal Sample Characteristics**

Table S2 below describe the sub-sample characteristics of the MT and NMT participants with available longitudinal data (total n = 98) and without longitudinal data (total n=77). As shown in Table S2, attrition at follow-up was substantial due to resource constraints, with follow-up prioritised for participants who completed the neuroimaging task at baseline. While this selection was driven by logistical feasibility, we examined whether participants with and without follow-up data differed systematically on key characteristics. Within each group (MT and NMT), there were no statistically significant differences between participants with and without follow-up data on the primary outcome – combined neurocognitive risk. Specifically, for the NMT group: χ²(2) = 4.40, p = 0.111 and for the MT group: χ²(2) = 0.44, p = 0.804. These results were further confirmed by bootstrapped confidence intervals (5,000 replicates) - NMT group: 95% BCa CI [0.083, 13.049]; MT group: 95% BCa CI [0.000, 1.544]. In addition, as can be seen in Table S2, comparisons of baseline symptoms and demographic variables (e.g., age, gender, pubertal status, ethnicity, IQ, and SES) showed no significant differences between participants with and without follow-up data, except for IQ, which was higher in the NMT group without follow-up (106.5) vs. with follow-up (102.0), p = 0.042, and socioeconomic status, which was higher in the MT group without follow-up (3.8) vs. with follow-up (3.1), p < 0.001. These differences, however, did not survive correction for multiple comparisons. Overall, these findings suggest no clear evidence of systematic attrition bias, although limitations in follow-up data availability should be considered when interpreting results.

| **Table S2**  Demographics, cognitive abilities, and psychological symptoms in maltreatment-exposed (MT) and non-maltreated (NMT) participants with either longitudinal (MT: n = 57; NMT: n = 41) or baseline-only (MT: n = 28; NMT: n = 49) data on symptoms | | | | | |
| --- | --- | --- | --- | --- | --- |
|  |  | MT  (longitudinal sample) | MT  (baseline-only sample) | NMT  (longitudinal sample) | NMT  (baseline-only sample) |
| *Measures* |  | Percentage | | Percentage | |
| Female | | 47.4% | 50.0% | 48.8% | 55.1% |
| Caucasian | | 35.1% | 46.4% | 53.7% | 36.7% |
|  |  | Mean (SD) | | Mean (SD) | |
| Age ^a^ |  | 12.8 (2.2) | 12.8 (2.7) | 12.8 (2.3) | 12.7 (2.0) |
| Pubertal status ^b^ | | 2.4 (0.9) | 2.3 (0.8) | 2.2 (0.8) | 2.5 (0.8) |
| WASI-IQ ^c^ | | 100.1 (9.9) | 95.3 (13.6) | 102.0 (9.0) | 106.5 (12.1) |
| Socio-economic status ^d^† | | 3.1 (0.9) | 3.8 (0.9) | 2.8 (0.9) | 3.1 (1.1) |
| Baseline SDQ total score ‡ ^e^* | | 12.0 (6.5) | 13.3 (6.2) | 7.9(5.0) | 6.6 (6.3) |
| Follow-up SDQ total score * | | 11.7 (6.8) | n/a | 9.1 (5.1) | n/a |
| * Sig. group difference between MT and NMT with longitudinal data only (not corrected for multiple comparisons).  † Sig. group difference within the MT group between those with only baseline data vs those with longitudinal data not corrected for multiple comparisons).  ‡ Sig. group difference within the NMT group between those with only baseline data vs those with longitudinal data not corrected for multiple comparisons).  ^a^ age range = 8-16 years. ^b^ Pubertal status was measured with the Puberty Development Scale (PDS) [7], ^c^ WASI-IQ = two IQ-subscales derived from the Wechsler Abbreviated Scales of Intelligence [8]. ^d^ Socio-economic status was measured using the highest parental/carer’s level of education rated on a 6-point scale from 0 (no formal qualifications) to 5 (postgraduate qualifications); ^e^ SDQ = Strength and Difficulties Questionnaire [9]. | | | | | |

**Tasks used to compute the ‘Cumulative Neurocognitive Vulnerability’ metric**

As already described in the main manuscript, the cumulative neurocognitive vulnerability metric was generated from participants’ performance across five behavioural experimental paradigms that assess neurocognitive functioning across domains of social cognition and reward processing. The paradigms included: (i) an adapted ‘Dot-Probe Task’ for the assessment of attention bias towards threat-related facial expressions; (ii) a trustworthiness face-judgement task to assess trust attributions towards unfamiliar faces; (iii) an adapted emotion recognition task to assess the perceived emotional intensity of dynamic facial expressions; (iv) the ‘Environmental Reward Learning Task’ to measure the ability to adjust contingency-based learning rates in stable and volatile environments; and (v) the ‘Apple Gathering Task’ to assess effort-based reward sensitivity. Below we will briefly outline the tasks that were used and, when appropriate, refer to published studies where the tasks are described in extenso.

***Threat-related attention bias.*** Published studies on the impact of childhood maltreatment on threat-processing have utilised the ‘Dot-Probe Task’ among children and young and reported attention bias for threat-related social cues [10–12]. In the present study, we used unpublished data from a dot-probe task in which participants were told to continuously look at a fixation cross in the centre of the screen, and then were shown two faces for 200ms and then two arrays of letters for 200ms. They were asked to respond as quickly as possible by pressing one of two keys, corresponding to the different sides of the screen, to identify which letter array contained the letter W. The faces shown were angry, calm, or neutral scrambled faces drawn from NimStim Face Stimulus Set [13]. In line with empirical recommendations for improving the stability of the dot-probe tasks and previous studies [12, 14], incorrect trials, along with those with latencies shorter than 200ms or longer than 2000ms, were discarded. For each participant, outlier trials (i.e. below or above 2 SD above each participant’s mean latency) were also removed. Participants had at least 75% of valid trials. Attention bias scores were computed by applying a commonly used formula [11, 12]. This involved subtracting the participant's average reaction time for trials in which the emotional face (threatening) and the probe appeared on the same side of the screen (congruent trials) from their average reaction time for trials where the emotional face and probe were on opposite sides (incongruent trials). The attention bias score for threat-related stimuli was the parameter of interest contributing towards the cumulative neurocognitive vulnerability metric.

***Trust bias***. The results of the trustworthiness face-judgement task have already been published elsewhere, showing that maltreatment-exposed participants show atypical trust attributions (more negative and more heterogeneous responses) [15]. We refer the reader to this article for a detailed description of the paradigm, the computational modelling that was used, as well as the robust validity and reliability properties of the task. Briefly, the task is employed to assess children's initial perceptions of trustworthiness towards unfamiliar faces. Participants were shown 70 facial stimuli individually and asked to respond ‘yes’ or ‘no’ to the question, ‘Is this face trustworthy?’ They indicated their responses by pressing a key for either ‘yes’ or ‘no’. There was no time limit imposed for responding to each trial. Each of the 70 trials began with a central fixation cross. Ten distinct face identities were randomly chosen from a pool of twenty-five computer-generated identities created using FaceGen Modeller 3.2 [16]. These ten identities had previously been modified by Todorov and colleagues (2013) to generate seven variations for each identity, with increments of one standard deviation ranging from -3 to +3 along a trustworthiness dimension [16]. The coloured facial images were presented in random order across the 70 trials. A ‘trust bias’ index was derived from the fitted psychometric functions denoting the level of face’s trustworthiness at which a participant was equally likely to respond ‘trustworthy’ or ‘not trustworthy’. Higher bias scores indicate less trusting judgement about novel/unfamiliar faces. The parameter of interest contributing to the cumulative vulnerability metric was the participants’ ‘trust bias’.

***Perceived emotional intensity***. The results of the adapted emotion recognition task that was used to assess the perceived emotional intensity are already published elsewhere [17] The results shows that maltreatment is associated with altered intensity perception of positive affect. We refer the reader to this article for a detailed description of the task. Briefly, participants viewed dynamic facial stimuli from the Amsterdam Dynamic Facial Expression Set – Bath Intensity Variations (ADFES-BIV) [18], which included videos showing three intensity levels of emotional expressions (low, medium, and high). Each video lasted 1040ms, starting with a neutral face and ending with the full expression. After each video, participants identified the emotion (joy, sadness, surprise, fear, or neutral) and rated its intensity on a scale of 1 (very little) to 10 (a lot). No intensity rating was required for neutral faces. Participants completed five practice trials before being presented with 24 randomised trials of happy and fearful faces (eight per intensity level). Additionally, 14 filler trials (neutral, sad, and surprise faces) were included to prevent response patterns but were not analysed. The parameter of interest used for the cumulative vulnerability metric was the average intensity scores for correctly identified positive (happy) faces, as this was the domain showing group differences and associations with symptoms.

***Reward-based learning.*** Contingency-based learning has consistently been associated with exposure to early adversity [19]. In this study, the learning component of reward processing (the ability to adjust and update outcome expectancies) was assessed using unpublished data (manuscript in preparation) from the ‘Environmental Reward Learning Task’. This task, adapted from Behrens and colleagues [20], evaluates learning of reward contingencies in both stable and unstable/volatile reward environments. Children were informed that they were playing for a real reward (vouchers valued up to £10). They were shown two pirates, each standing beside a treasure chest with a flag displaying randomly chosen reward values between 0 and 100, with a combined total of 100. They were told that only one chest held the gold coins indicated by the flag (the rewarded stimulus), while the other was empty regardless of the flag’s value (the non-rewarded stimulus). Children had to decide which pirate they believed had the gold coins by pressing a key before the reward was revealed, earning points for correct choices. The reward structure was not explained, and they were only instructed to aim for winning the gold coins each time to reach the highest level. The task consisted of 180 trials split into two conditions: in the stable condition, the red pirate was rewarded at a 75:25 ratio across 80 trials, while in the volatile condition, the reward ratio shifted between 80:20 and 20:80 every 20 trials. The 180 trials were presented in one session, with the order of conditions (stable or volatile) counterbalanced across participants. Some participants completed the fMRI version of this task, which was slightly shorter – 120 trials. During the task, if children chose the rewarded stimulus, their points increased based on the flag's value, represented by a bar chart on the screen, with visual feedback (“Well done, you chose correctly”) and a sound of coins dropping. Incorrect choices resulted in the message “Better luck next time” with no change in the bar chart. When the bar was filled, children were informed they had reached a new level, and the game continued. At the end, they were told their final level and points total, e.g., “You reached level 10 and collected 1500 points.” All participants were assured they had won enough points to receive a monetary reward. The parameter, used towards the cumulative neurocognitive vulnerability metric calculation, was the computationally-derived overall “learning rate”. This reflects how recent information/feedback (i.e. prediction error) correctly influences the updating of associative values (i.e. expected value representations).

***Effort-based reward sensitivity***. Effort-based decision-making for reward was evaluated using the ‘Apple Gathering Task’. The findings from this task, which have been published in [21], revealed atypical effort-based reward sensitivity in participants exposed to maltreatment. For a comprehensive description of the task and the computational modelling that was used, we direct readers to that article. Briefly, during this task children were told they were playing for a real reward (a voucher for an online shop). The task used a grip squeeze device (hand dynamometer) to measure physical effort exerted for a reward (apples falling from a tree). The actual amount of force required was calibrated on an individual basis so that the difficulty of the task was equivalent across participants, irrespective of physical strength. To win the apples participants had to grip the squeeze device to “shake” the tree. Participants could either accept the effort challenge and play the trial or skip to the next trial if they did not feel that the potential reward was worth the effort. There were 3 blocks of a total of 72 trials with 3 different effort and 3 different reward levels. The main outcome parameter, used to compute the cumulative neurocognitive vulnerability metric, was a computationally derived parameter for the individual sensitivity to effort-based reward.

**Cumulative Neurocognitive Vulnerability Metric**

***Threshold for normative vs. atypical classification***. For each task, values beyond ±3 standard deviations from the overall sample mean were excluded prior to applying the normative/atypical classification threshold. This ensured that extreme scores, potentially driven by artefactual or non-representative responding, did not disproportionately influence classification. This step removed only task-level data points (not participants), and participants were retained in the analysis provided they had usable data for at least two tasks. After trimming extreme values, we classified participants' performance on each task as either *normative* (score = 0; within 1 SD from the sample overall mean) or *atypical* (score = 1; more than 1 SD below or above the mean). The choice of 1 SD as the threshold aligns with common practices in cognitive and psychological research, where deviations beyond this point often reflect meaningful differences in performance compared to the normative range [22–24]. Scores falling more than 1 SD from the mean can indicate atypical neurocognitive functioning, suggesting potential difficulties or altered processing in the domains of interest (e.g. reward sensitivity, attention bias). By using this criterion, we aim to capture participants who exhibit significant deviations from the average, which may correspond to heightened neurocognitive vulnerability.

Maltreatment has consistently been associated with a blunted neurocognitive response during contingency-based learning tasks [19, 25]. Conversely, higher neurocognitive responses and sensitivity to rewarding/affiliative social stimuli have been associated with resilient functioning and outcomes [17, 26, 27]. Therefore, scores *below* 1SD were considered atypical (i.e. denoting potential neurocognitive vulnerability) for the ‘reward sensitivity’ scores in the Apple Gathering Task, ‘learning rate’ scores in the Environmental Reward Learning Task, and ‘perceived intensity’ scores for positive affect in the emotion recognition task.

In the current literature, maltreatment exposure has been shown to have a non-linear relationship with both threat-related neurocognitive alterations and trust attributions. This means that rather than a straightforward increase or decrease in neurocognitive responses based on the level of maltreatment exposure, the relationship may involve complex patterns. For example, some studies suggest that individuals exposed to maltreatment may show both neurocognitive avoidance (e.g., avoiding threat-related stimuli) and hypervigilance (i.e. heightened sensitivity to detecting threat), depending on the specific context or severity of the maltreatment [10, 11, 28]. Similarly, trust behaviours in maltreated individuals may not follow a linear decline; rather, they might exhibit diminished trust towards unfamiliar individuals but, in some cases, display disinhibited or excessive trust in inappropriate contexts [29–36]. Therefore, considering the non-linearity of current empirical findings, for the parameters ‘trust bias’ in the Trustworthiness Face-Judgement task and ‘attention bias’ in the Dot-Probe task, values both *above* and *below* 1 SD from the mean were considered atypical (i.e. denoting potential neurocognitive vulnerability).

***Summary of atypical classification criteria (by task).* *Emotion recognition, reward sensitivity, and reward-based learning:*** Scores >1 SD ***below*** the mean were considered atypical. ***Threat attention-bias and trust bias*:** Due to established non-linear patterns in maltreatment-related responses, scores >1 SD ***above or below*** the mean were considered atypical.

***Trinary cumulative neurocognitive vulnerability***. Subsequently, for those participants who completed and had usable data on at least two of the five tasks (see Supplementary Table S5 for a detailed count), the binary scores from each task (i.e. 0 = normative, 1 = atypical) were used to compute a trinary cumulative neurocognitive vulnerability metric. As can be seen in Table 2 in the main manuscript, 86 participants, who had a normative score on all available tasks, were classified as low-vulnerability (i.e. trinary cumulative vulnerability score = 0); 65 participants, who had an atypical score on only one task, were classified as medium-vulnerability (i.e. trinary cumulative vulnerability score = 1); and 24 participants, who showed atypical scores on two (n = 18) or three tasks (n = 6), were classified as high-vulnerability (i.e. trinary cumulative vulnerability score = 2).

**Maltreatment Severity and Subtype – Kaufman Scale**

| **Table S3**  *Maltreatment Subtype Severity in the MT Group* | | | | |
| --- | --- | --- | --- | --- |
| Maltreatment Subtype | N | % | Mean Severity^1^ (1-4) | SD |
| Neglect | 58 | 68.2 | 3.8 | 2.2 |
| Physical Abuse | 20 | 23.5 | 1.4 | 0.7 |
| Sexual Abuse | 9 | 10.6 | 2.1 | 1.3 |
| Emotional Maltreatment | 77 | 90.6 | 2.5 | 1.1 |
| Home Violence | 70 | 82.4 | 1.5 | 0.9 |
| ^1^Maltreatment severity scores were calculated based on Kaufman Scale [37] | | | | |

The Kaufman scale [37] was used to measure the prevalence of maltreatment subtypes and severity, and degree of polyvictimsiation among the group of children and adolescents with corroborated maltreatment exposure (MT Group, shown in Table S3). This tool assesses maltreatment subtypes, including neglect, physical abuse, sexual abuse, emotional maltreatment, and home violence exposure. Each category of childhood maltreatment is rated from zero to four. The scales’ Kappa reliability coefficient ranges between.73 and .90. In terms of validity, the authors found that maltreatment ratings were significantly associated with externalising symptoms (r =.57; p<.001) and internalising symptoms (r=.29, p =.03). In terms of inter-rater reliability, the authors noted that “there were no discrepancies between raters that were greater than one scale point”. The degree of exposure to multiple forms of maltreatment in our sample, according to official records, was a follow: 4 individuals experience one form of maltreatment, 31 experiences two, 29 experienced three, 13 experienced four, and 4 experienced five types. That is, most of the individuals in the MT group had experienced polyvictimisation during childhood.

**Supplementary Results**

**Childhood maltreatment and cumulative neurocognitive vulnerability**

The contingency table below (Table S4) provide an overview of the cumulative neurocognitive vulnerability levels (low, medium and high) by maltreatment group (MT vs. NMT) after applying the propensity score matching (PSM) weighting variable. The significant bootstrapped chi-square test [χ² = 1.1, p < .05 (95% CI = 0.3 – 9.8)], reported in the main manuscript, indicate that group difference on demographic variable, did not influence the group difference on the cumulative neurocognitive vulnerability index, with participants in the MT group being more likely to fall into higher vulnerability categories compared to those in the NMT group, even after PSM.

| **Table S4**  *Contingency table of cumulative neurocognitive vulnerability levels (low, medium and high) by maltreatment group (MT vs. NMT) after propensity score matching (PSM)* | | | | |
| --- | --- | --- | --- | --- |
|  | Low vulnerability | Medium vulnerability | High vulnerability | Rows Total |
| NMT group | 53.6 (59.6%) | 30.9 (34.3%) | 5.5 (6.1%) | 90 (100%) |
| MT group | 34 (40.0%) | 36 (42.4%) | 15 (17.6%) | 85 (100%) |
| Columns Total | 87.6 | 66.9 | 20.5 |  |
| *Note:* Values are adjusted by the PSM weighting variable. Percentage values are expressed as a proportion of the row’s totals. | | | | |

**Number of tasks used to estimate the cumulative neurocognitive vulnerability index.** As described in the main manuscript, participants were included in this study if they had available data on at least two of the five tasks that were used to estimate the cumulative neurocognitive vulnerability metric. As shown in Table S5 below, the two groups showed a significant difference in the number of available tasks (χ² = 15.1, df = 3, p = 0.002), with more MT participants having data available on four or five tasks. This could have introduced a group bias in relation to the cumulative neurocognitive vulnerability index, as a greater number of available tasks, could mean a greater probability of having a higher cumulative neurocognitive vulnerability score. To assess the existence of such potential bias, we run a Kendall’s Tau analysis (i.e. appropriate for examining the association between two ordinal variables) to measure the correlation between the number of available tasks and the cumulative neurocognitive vulnerability score. We found this to be non-significant in both the NMT (τ = .15, p = .12) and MT groups (τ = -.03 p = .75). This suggests that, for both group, there is no evidence of an association between the number of tasks available and neurocognitive vulnerability scores.

| **Table S5**  *Contingency table of participant counts by Group (MT vs. NMT) for the number of available tasks used to calculate the cumulative neurocognitive vulnerability* *metric.* | | | |
| --- | --- | --- | --- |
| N^o^ of available tasks | MT | NMT | Total |
| 2 | 13 | 28 | 41 |
| 3 | 13 | 25 | 38 |
| 4 | 39 | 28 | 67 |
| 5 | 20 | 9 | 29 |

Furthermore, we run an ordinal logistic regression, to assess if the group difference on the cumulative neurocognitive vulnerability metric remained significant after controlling for the number of available tasks. As shown in Table S6, we found that being the MT group significantly increases the likelihood of fall into a higher neurocognitive vulnerability category, even after controlling for the number of available tasks (which were not statistically associated with the cumulative neurocognitive vulnerability index). Therefore, the higher neurocognitive vulnerability scores in the MT group does not seem to be driven (biased) by the amount of available tasks.

| **Table S6**  *Ordinal logistic regression results showing the association between group status (MT vs NMT) and cumulative neurocognitive vulnerability* *levels (low, medium and high) after controlling for the number of available tasks.* | | | | | |
| --- | --- | --- | --- | --- | --- |
|  | B | Bias | SE | 95% CI | Odds Ratio (OR) |
| Group (MT) ***** | 0.7 | 0.0 | 0.3 | [0.1, 1.3] | 2.0 |
| Total Available Tasks (L) | 0.2 | 0.0 | 0.3 | [-0.5, 0.9] | 1.6 |
| Total Available Tasks (Q) | -0.4 | -0.0 | 0.3 | [1.0, 0.2] | 0.4 |
| Total Available Tasks (C) | 0.0 | -0.0 | 0.3 | [-0.6, 0.6] | 1.0 |
| **sig. bootstrapped predictors*  *Note:* The main independent variable (‘Group) has two categories, MT = maltreatment exposed group and NMT = non-maltreatment exposed group (i.e. the reference category). The ordinal dependent variable, ‘Cumulative Neurocognitive Vulnerability, has three levels: low, medium and high. The coefficients of the ordinal covariate ‘Total Available Tasks’ are described in terms of their Linear (L), quadratic (Q) and cubic (C) effects. Results are obtained using bootstrapping with 5000 repetitions. The coefficients (B) represent the log odds. Bias is the difference between the bootstrapped estimate and the original coefficient. | | | | | |

**MT Group**

**Cumulative neurocognitive vulnerability and longitudinal symptoms change in the MT group**

Table 2 in the main manuscript reports the full results of the logistic regression performed to measure if cumulative neurocognitive vulnerability score in the MT group is predictive of future symptoms change, after controlling for baseline symptoms levels (i.e. SDQ total score). As reported there, it shows that being in the high-vulnerability category is a significant predictor of future symptoms increase, independently of baseline symptoms levels (Table 2).

| **Table S7**  *Contingency table of symptoms change (stable/decrease vs increase) by cumulative neurocognitive vulnerability* *levels (low, medium and high) in the maltreatment exposed group (MT)* | | | |
| --- | --- | --- | --- |
|  | Stable/decrease | Increase | Rows total |
| Low vulnerability | 15 (65.2%) | 8 (34.8%) | 23 (100%) |
| Medium vulnerability | 14 (60.9%) | 9 (39.1%) | 23 (100%) |
| High vulnerability | 1 (9.1%) | 10 (90.9%) | 11 (100%) |
| Columns total | 30 | 27 |  |

Statistical testing without baseline symptoms scores as a covariate also yielded results in the same direction. The bootstrapped (R = 5000) chi-square test results indicated significant differences in the distribution of cumulative neurocognitive vulnerability scores (low-, medium-, and high-vulnerability) at baseline as a function of individuals who longitudinally showed increased vs. decreased/stable symptoms; χ² = 10.5, bias = 1.5, SE = 5.0, p < .05 (95% CI = 1.6, 19.7). Being in the neurocognitive high-vulnerability category at baseline was associated with an increased probability of experiencing symptoms increase at follow-up (OR = 10.0), while being in the low- (OR = 0.5) and medium-vulnerability (OR = 0.6) categories was associated with an increased probability of decreased or stable symptoms at follow-up (Table S7).

***Model’s assumptions and model fit****.* Models’ collinearity assumption was met, as shown by low Variance Inflation Factor (VIF; Cumulative Vulnerability Score GVIF adjusted = 1.0, Baseline Symptoms GVIF adjusted = 1.0). The Hosmer-Lemeshow test had a p-value of 0.32, indicating a good fit of the model to the data. The logistic regression model's goodness of fit was also assessed using several pseudo R-squared measures. The McFadden’s R-squared value 0.17 indicates a moderate fit. Cragg & Uhler’s R-squared (Nagelkerke’s R-squared) was 0.27, suggesting also a good fit. McKelvey & Zavoina's R-squared was 0.20, further supporting a good fit of the model. The log-likelihood of the fitted model was -32.9, and the log-likelihood of the null model was -39.4, resulting in a likelihood ratio chi-square (G2) of 13.0, indicating a significant improvement over the null model. Finally, Cook's Distance was calculated to assess the influence of individual data points on the logistic regression model. Four data points (participants) were identified as potentially influential. After excluding these points, the logistic regression model was refitted, and no significant changes were observed in the regression coefficients or overall model fit. This indicates that the model is robust and not unduly influenced by these data points.

***Sensitivity Analyses.*** As shown in Table S8 below, after removing 8 MT participants whose baseline scores were abnormally elevated (≥ 20 “very high” SDQ four-band categorisation), being in the neurocognitive high-vulnerability group, still represented a significant predictor of future symptoms increase. In other words, the prognostic value of the cumulative neurocognitive vulnerability index does not seem to be driven by participants who, at baseline, may already by presenting with a frank psychopathology.

| **Table S8**  *Logistic regression results predicting symptoms change (decrease/stable vs increase) from cumulative neurocognitive vulnerability levels (low, medium and high) and baseline symptom levels (SDQ baseline total score) in the MT group, after participants with SDQ baseline scores* ≥ 20 were removed | | | | | |
| --- | --- | --- | --- | --- | --- |
|  | B | Bias | SE | 95% CI | Odds Ratio (OR) |
| Intercept | 0.8 | 0.1 | 1.2 | [-0.8, 3.5] | 2.3 |
| Medium vulnerability (ref: low vulnerability) | 0.3 | 0.0 | 1.2 | [-1.5, 2.1] | 1.3 |
| High vulnerability (ref: low vulnerability) ***** | 3.5 | 5.9 | 8.1 | [1.6, 22.0] | 34.0 |
| Baseline Symptoms ***** | -0.2 | -0.0 | 0.1 | [-0.4, -0.1] | 0.8 |
| **sig. bootstrapped predictors*  *Note:* Cumulative neurocognitive vulnerability is an ordinal variable with three levels: low, medium and high. The low-vulnerability category serves as the reference group. Results are obtained using bootstrapping with 5000 repetitions. The coefficients (B) represent the log odds. Bias is the difference between the bootstrapped estimate and the original coefficient. | | | | | |

**Cumulative neurocognitive vulnerability, symptoms severity and maltreatment severity in the MT group**

We explored if the baseline cumulative neurocognitive vulnerability metric was associated (cross-sectionally) with baseline symptoms levels (i.e. SDQ total score) and with maltreatment severity (i.e. Kaufman scale total score) [37]. The Kendall’s Tau analysis revealed non-significant associations between the neurocognitive vulnerability metric with baseline symptoms (τ = -.04, p = .69) and maltreatment severity (τ = .09, p = .31). These results suggest that, among children and adolescents exposed to substantiated abuse and neglect, cumulative neurocognitive vulnerability is not associated with concurrent symptoms nor maltreatment severity.

**NMT Group**

**Cumulative neurocognitive vulnerability and longitudinal symptoms change in the NMT group**

The tables below (Table S9 and Table S10) report the contingency table and full results of the logistic regression performed to measure if cumulative neurocognitive vulnerability score in NMT group is predictive of future symptoms change, after controlling for baseline symptoms levels (i.e. SDQ total score). As reported in the main manuscript, it shows that, among the group of children and young people without substantiated exposure to childhood maltreatment, the cumulative neurocognitive vulnerability metric was not found to be a significant predictor of future symptoms change (i.e. non-significant predicted probability - Figure 1S).

**Figure 1S**

*Predicted probability of longitudinal symptoms increase by neurocognitive vulnerability categories (low, medium and high) in maltreatment-exposed participants (MT group).*


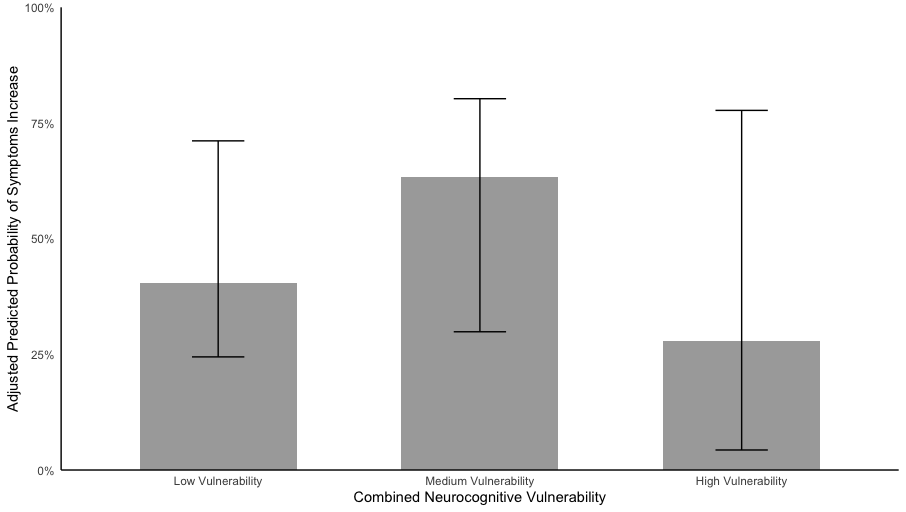


Note: Error bars represent 95% Clopper-Pearson confidence intervals within each vulnerability category. The Predicted Probability values are adjusted for baseline symptom levels.

| \| **Table S9**  *Contingency table of symptoms change (stable/decrease vs increase) by cumulative neurocognitive vulnerability levels (low, medium and high) in the non-maltreatment exposed group (NMT)* \| \| \| \| \| --- \| --- \| --- \| --- \| \|  \| Stable/decrease \| Increase \| Rows total \| \| Low vulnerability \| 10 (52.6%) \| 9 (47.4%) \| 19 (100%) \| \| Medium vulnerability \| 7 (43.8%) \| 9 (56.2%) \| 16 (100%) \| \| High vulnerability \| 4 (66.7%) \| 2 (33.3%) \| 6 (100%) \| \| Columns total \| 21 \| 20 \|  \|   **Table S10**  *Logistic regression results predicting symptoms change (decrease/stable vs increase) from cumulative neurocognitive vulnerability levels (low, medium and high) and baseline symptom levels (SDQ baseline total score) in the NMT group* | | | | | |
| --- | --- | --- | --- | --- | --- | --- | --- | --- | --- | --- | --- | --- | --- | --- | --- | --- | --- | --- | --- | --- | --- | --- | --- | --- | --- | --- | --- | --- | --- |
|  | B | Bias | SE | 95% CI | Odds Ratio (OR) |
| Intercept | 1.2 | -0.9 | 0.7 | [-0.9,1.7] | 3.3 |
| Medium vulnerability (ref: low vulnerability) | 0.9 | -0.7 | 0.8 | [-0.4, 0.9] | 2.5 |
| High vulnerability (ref: low vulnerability) | -0.6 | -3.2 | 1.0 | [-16.1, 1.3] | 0.6 |
| Baseline Symptoms | -0.2 | -3.9 | 0.1 | [-16.5, 1.0] | 0.8 |
| *Note:* Cumulative neurocognitive vulnerability is an ordinal variable with three levels: low, medium and high. The low-vulnerability category serves as the reference group. Results are obtained using bootstrapping with 5000 repetitions. The coefficients (B) represent the log odds. Bias is the difference between the bootstrapped estimate and the original coefficient. | | | | | |

***Model’s assumptions and model fit.*** Model's collinearity assumptions were met, as shown by low Variance Inflation Factor (VIF) values (cumulative vulnerability score GVIF adjusted = 1.0, baseline symptoms GVIF adjusted = 1.1). The Hosmer-Lemeshow test had a p-value of 0.64, indicating a good fit of the model to the data. The logistic regression model's goodness of fit was also assessed using several pseudo R-squared measures. The McFadden’s R-squared value was 0.14, indicating a moderate fit. Cragg & Uhler’s R-squared (Nagelkerke’s R-squared) was 0.23, suggesting a relatively good fit. McKelvey & Zavoina's R-squared was 0.17, further supporting a good fit of the model. The log-likelihood of the fitted model was -24.6, and the log-likelihood of the null model was -28.4, resulting in a likelihood ratio chi-square (G2) of 7.7, indicating a significant improvement over the null model. Finally, Cook's Distance was calculated to assess the influence of individual data points on the logistic regression model. Three data points (participants) were identified as potentially influential. After excluding these points, the logistic regression model was refitted, and no significant changes were observed in the regression coefficients or overall model fit. This indicates that the model is robust and not unduly influenced by these data points.

**References**

[1] McCaffrey DF, Ridgeway G, Morral AR. Propensity score estimation with boosted regression for evaluating causal effects in observational studies. *Psychol Methods* 2004; 9: 403–425.

[2] Ho DE, Imai K, King G, et al. Matchit : Nonparametric preprocessing for parametric causal inference. *J Stat Softw* 2011; 42: 1–28.

[3] Ho DE, Imai K, King G, et al. Matching as nonparametric preprocessing for reducing model dependence in parametric causal inference. *Political Analysis* 2007; 15: 199–236.

[4] Stuart EA. Matching Methods for Causal Inference: A Review and a Look Forward. *Statistical Science* 2010; 25: 1–21.

[5] Pingault JB, Cote SM, Petitclerc A, et al. Assessing the independent contribution of maternal educational expectations to children’s educational attainment in early adulthood: A propensity score matching analysis. *PLoS One*; 10. Epub ahead of print 2015. DOI: 10.1371/journal.pone.0119638.

[6] Stuart EA, Lee BK, Leacy FP. Prognostic score-based balance measures can be a useful diagnostic for propensity score methods in comparative effectiveness research. *J Clin Epidemiol*; 66. Epub ahead of print 2013. DOI: 10.1016/j.jclinepi.2013.01.013.

[7] Petersen AC, Crockett L, Richards M, et al. A self-report measure of pubertal status: Reliability, validity, and initial norms. *J Youth Adolesc* 1988; 17: 117–133.

[8] Wechsler D. *WASI-II: Wechsler abbreviated scale of intelligence*. 2nd ed. San Antonio, TX: Psychological Corporation, 2011.

[9] Malecki CK, Demaray MK. Measuring perceived social support: Development of the child and adolescent social support scale (CASSS). *Psychol Sch* 2002; 39: 1–18.

[10] Pollak SD, Tolley-Schell S a. Selective attention to facial emotion in physically abused children. *J Abnorm Psychol* 2003; 112: 323–338.

[11] Pine DS, Mogg K, Bradley BP, et al. Attention bias to threat in maltreated children: implications for vulnerability to stress-related psychopathology. *Am J Psychiatry* 2005; 162: 291–6.

[12] Kelly PA, Viding E, Puetz VB, et al. Sex differences in socioemotional functioning, attentional bias, and gray matter volume in maltreated children: A multilevel investigation. *Dev Psychopathol* 2015; 27: 1591–1609.

[13] Tottenham N, Tanaka JW, Leon AC, et al. The NimStim set of facial expressions: Judgments from untrained research participants. *Psychiatry Res*; 168. Epub ahead of print 2009. DOI: 10.1016/j.psychres.2008.05.006.

[14] Price RB, Kuckertz JM, Siegle GJ, et al. Empirical recommendations for improving the stability of the dot-probe task in clinical research. *Psychol Assess* 2015; 27: 365–376.

[15] Neil L, Viding E, Armbruster-Genc D, et al. Trust and childhood maltreatment: evidence of bias in appraisal of unfamiliar faces. *J Child Psychol Psychiatry* 2022; 63: 655–662.

[16] Todorov A, Dotsch R, Porter JM, et al. Validation of data-driven computational models of social perception of faces. *Emotion* 2013; 13: 724–738.

[17] Gerin MI, Viding E, Neil L, et al. Heightened response to positive facial cues as a potential marker of resilience following childhood adversity. *Eur J Psychotraumatol*; 15. Epub ahead of print 2024. DOI: 10.1080/20008066.2024.2309783.

[18] Wingenbach TSH, Ashwin C, Brosnan M. Validation of the Amsterdam Dynamic Facial Expression Set ’ Bath Intensity Variations (ADFES-BIV): A Set of Videos Expressing Low, Intermediate, and High Intensity Emotions. *PLoS One*; 11. Epub ahead of print 2016. DOI: 10.1371/journal.pone.0147112.

[19] Oltean LE, Șoflău R, Miu AC, et al. Childhood adversity and impaired reward processing: A meta-analysis. *Child Abuse Negl*; 142. Epub ahead of print 2023. DOI: 10.1016/j.chiabu.2022.105596.

[20] Behrens TEJ, Woolrich MW, Walton ME, et al. Learning the value of information in an uncertain world. *Nat Neurosci* 2007; 10: 1214–1221.

[21] Armbruster-Genç DJN, Valton V, Neil L, et al. Altered reward and effort processing in children with maltreatment experience: a potential indicator of mental health vulnerability. *Neuropsychopharmacology* 2022; 1–8.

[22] Busse A, Hensel A, Gühne U, et al. Mild cognitive impairment: Long-term course of four clinical subtypes. *Neurology*; 67. Epub ahead of print 2006. DOI: 10.1212/01.wnl.0000249117.23318.e1.

[23] Chen YC, Tsai WH, Ho CH, et al. Atypical sensory processing and its correlation with behavioral problems in late preterm children at age two. *Int J Environ Res Public Health*; 18. Epub ahead of print 2021. DOI: 10.3390/ijerph18126438.

[24] McDonald CR, Busch RM, Reyes A, et al. Development and Application of the International Classification of Cognitive Disorders in Epilepsy (IC-CoDE): Initial Results From a Multi-Center Study of Adults With Temporal Lobe Epilepsy. *Neuropsychology*; 37. Epub ahead of print 2022. DOI: 10.1037/neu0000792.

[25] Gerin MI, Hanson E, Viding E, et al. A review of childhood maltreatment, latent vulnerability and the brain: implications for clinical practice and prevention. *Adopt Foster* 2019; 43: 310–328.

[26] Dennison MJ, Sheridan MA, Busso DS, et al. Neurobehavioral markers of resilience to depression amongst adolescents exposed to child abuse. *J Abnorm Psychol* 2016; 125: 1201–12012.

[27] Hanson JL, Albert D, Iselin AMR, et al. Cumulative stress in childhood is associated with blunted reward-related brain activity in adulthood. *Soc Cogn Affect Neurosci* 2016; 11: 405–412.

[28] Hoepfel D, Günther V, Bujanow A, et al. Experiences of maltreatment in childhood and attention to facial emotions in healthy young women. *Sci Rep*; 12. Epub ahead of print 2022. DOI: 10.1038/s41598-022-08290-1.

[29] Gobin RL, Freyd JJ. The impact of betrayal trauma on the tendency to trust. *Psychol Trauma* 2014; 6: 505–511.

[30] Sharp C, Fonagy P. Social cognition and attachment-related disorders. In: *Social Cognition and Developmental Psychopathology*. 2013, pp. 271–302.

[31] Baer J, Martinez CD. Child maltreatment and insecure attachment: A meta-analysis. *Journal of Reproductive and Infant Psychology* 2006; 24: 187–197.

[32] Fonagy P, Allison E. The Role of Mentalizing and Epistemic Trust in the Therapeutic Relationship. *Psychotherapy* 2014; 51: 372–380.

[33] Eslinger J, Sprang G, Jodts J. Keeping children and youth in trauma treatment: Examination of an alliance building dropout management program. *Clin Child Psychol Psychiatry* 2023; 28: 721–733.

[34] Pitula CE, Wenner JA, Gunnar MR, et al. To trust or not to trust: social decision-making in post-institutionalized, internationally adopted youth. *Dev Sci*; 20. Epub ahead of print 2017. DOI: 10.1111/desc.12375.

[35] Kay C, Green J. Reactive attachment disorder following early maltreatment: Systematic evidence beyond the institution. *J Abnorm Child Psychol*; 41. Epub ahead of print 2013. DOI: 10.1007/s10802-012-9705-9.

[36] Zeanah CH, Gleason MM. Annual research review: Attachment disorders in early childhood - Clinical presentation, causes, correlates, and treatment. *J Child Psychol Psychiatry* 2015; 56: 207–222.

[37] Kaufman J, Jones B, Stieglitz E, et al. The use of multiple informants to assess children’s maltreatment experiences. *J Fam Violence* 1994; 9: 227–248.
